# Anticancer effect of tectochrysin in colon cancer cell via suppression of NF-kappaB activity and enhancement of death receptor expression

**DOI:** 10.1186/s12943-015-0377-2

**Published:** 2015-06-30

**Authors:** Mi Hee Park, Ji Eun Hong, Eun Sook Park, Hee Sung Yoon, Doo Won Seo, Byung Kook Hyun, Sang-Bae Han, Young Won Ham, Bang Yeon Hwang, Jin Tae Hong

**Affiliations:** College of Pharmacy and Medical Research Center, Chungbuk National University, 194-31 Osongsaengmyeong 1-ro, Osong-eup, Heungdeok-gu, Cheongju, Chungbuk 361-951 Republic of Korea; Department of Chemistry and Biochemistry, Brigham Young University, Provo, UT USA

**Keywords:** Colon cancer, Tectochrysin, NF-kappaB, Death receptor, Apoptosis

## Abstract

**Background:**

Flavonoids are a diverse family of natural phenolic compounds commonly found in fruits and vegetables. Epidemiologic studies showed that flavonoids also reduce the risk of colon cancer. Tectochrysin is one of the major flavonoids of *Alpinia oxyphylla* Miquel. However, the anti-cancer effects and the molecular mechanisms of tectochrysin in colon cancer cells have not yet been reported. We investigated whether tectochrysin could inhibit colon cancer cell growth at 1, 5, 10 μg/ml. In *in vivo* study, we injected a tectochrysin treatment dose of 5 mg/kg to each mouse.

**Results:**

Tectochrysin suppressed the growth of SW480 and HCT116 human colon cancer cells. The expression of DR3, DR4 and Fas were significantly increased, and pro-apoptotic proteins were also increased. Tectochrysin treatment also inhibited activity of NF-κB. A docking model indicated that tectochrysin binds directly to the p50 unit. In *in vivo*, tumor weights and volumes in mice were reduced when treated with tectochrysin. Tectochrysin leads to apoptotic cell death in colon cancer cells through activation of death receptors expression via the inhibition of NF-κB.

**Conclusions:**

Tectochrysin can be a useful agent for the treatment of colon cancer cell growth as well as an adjuvant agent for chemo-resistant cancer cells growth.

## Background

Colon cancer is the third most commonly diagnosed cancers in the world [1], with an estimated 140,000 new cases and over 50,830 deaths in 2013 in the USA, and over 1.2 million new cases and 600,000 deaths worldwide [2]. Surgery, radiotherapy and chemotherapy are used for treating colon cancer patients [3]. However, those treatments are not sufficient because of resistance against chemotherapy, toxicity and side-effects. Therefore, it is urgent to develop novel anti-cancer agents with fewer side effects to satisfy the unfulfilled therapeutic demands of patients.

Apoptosis plays an important role in anti-cancer effects of chemotherapeutics [4]. Apoptosis can be induced by stimulation of death receptor (DRs) [5]. Binding to their respective ligands, DRs are activated and recruit the intracellular adaptor protein (Fas-associated death domain protein) which results in the activation of caspase-3, caspase-8 and caspase-9 as well as Bax to kill cancer cells [6]. Chemo-resistances are also related to the expression of DRs in colon cancer cells [7]. Therefore, DR-mediated apoptosis has emerged as an effective strategy for cancer therapy. Several compounds have demonstrated the anticancer effects through activation of DRs. Baicalein, a naturally occurring flavonoid, enhanced TRAIL-induced apoptosis via increase of DR5 expression [8]. Casticin, a flavonoid, induced apoptosis through stimulation of the expression of DRs in colon cancer cells [9]. Nuclear factor-κB (NF-κB) represents a family of eukaryotic transcription factors participating in the regulation of various cellular responses involved in apoptosis [10]. NF-κB plays a regulatory role in the expression of an array of apoptotic (caspase-3 and Bax), anti-apoptotic (Bcl-2 and IAP family), and cell proliferation gens (cylooxygenase-2 and cyclins) [11]. NF-κB is constitutively activated in human colorectal carcinoma tissue and colon cancer cells [12]. From this knowledge, NF-κB can be specifically targeted to prevent colon cancer cell growth. Several of our studies have demonstrated that compounds inhibiting NF-κB have shown to be useful for inhibition of colon cancer cell growth. 4-O-methylhonokiol and inflexinol inhibited colon cancer cell growth through suppression of NF-κB pathway [13, 14]. Furthermore, the activation of NF-κB in response to chemotherapy is a principal mechanism of inducible chemo-resistance [15–18]. Thus, inactivation of NF-κB is intended as a strategy to eliminate cancerous cells.

Flavonoids are a diverse family of natural phenolic compounds commonly found in fruits and vegetables [19]. It is classified as flavonols, flavonones, flavans, etc. Flavonoids especially display a wide range of pharmacological properties including anti-inflammatory, anti-mutagenic, anti-carcinogenic and anti-cancer effects [20]. An epidemiologic study also showed that flavonoids reduce the risk of colon cancer [21]. Several studies have reported that some flavonoids have direct effects on apoptosis in colon cancer cell. Luteolin, a type flavonoid, has effects on apoptosis of colon cancer cells [22]. Another flavonoid, chrysin, also suppressed cancer cell growth through inhibition of the expression of NF-κB in colon cancer cells. *Alpinia oxyphylla* Miquel is used for treating intestinal disorders, dieresis, uresis, ulceration and diarrhea [23]. Yakuchinone A and yakuchinone B existed in *Alpinia oxyphylla* Miquel (Zingiberaceae) have anti-cancer effects in skin carcinogenesis [24]. Tectochrysin, another flavonoid compound, is isolated from *Alpinia oxyphylla* Miquel*.* Our previous study showed that tectochrysin suppressed lung cancer cell growth via inactivation of STAT3 [25]. Moreover, our preliminary study showed that tectochrysin was found to bind NF-κB. However, the anti-cancer effects and the molecular mechanisms of tectochrysin in colon cancer cells have not yet been reported. Thus, in this study, we investigated whether tectochrysin could inhibit colon cancer cell growth via suppression of NF-κB activity and enhancement of DR expression in *in vitro* and *in vivo*.

## Results

### Effect of tectochrysin on cell growth and apoptosis cell death

**Fig. 1:**
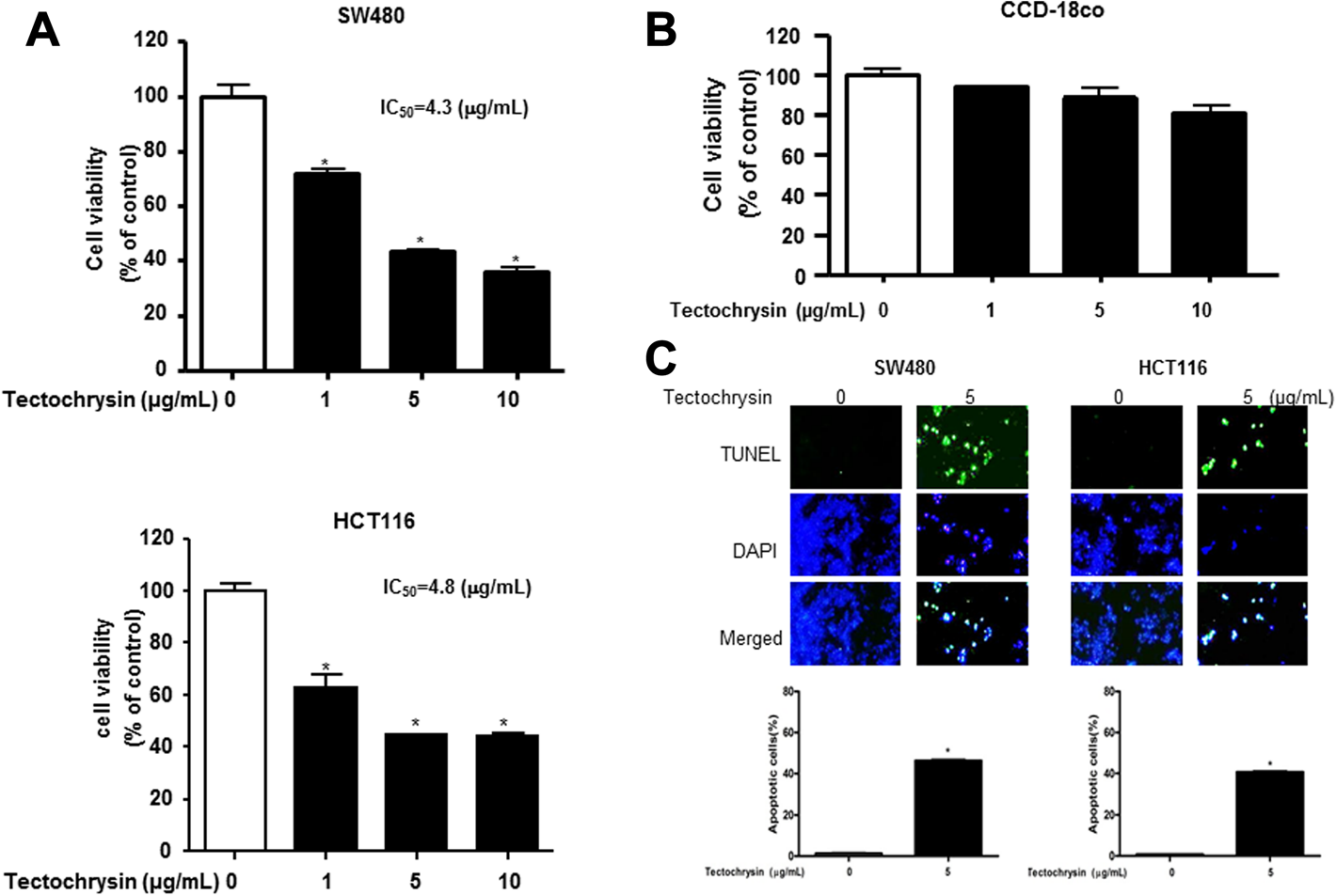
Effect of tectochrysin on cell growth and apoptotic cell death in colon cancer cells. **a** Concentration-dependent effect of tectochrysin on the cell growth in SW480 and HCT116 cells. After treatment of tectochrysin (1, 5 and 10 μg/mL) for 72 h, the effect of tectochrysin on colon cancer cell growth was determined by MTT assay and morphologic observation. **b** Concentration-dependent cell growth inhibitory effect of tectochrysin in CCD-18co normal colon cell. After treatment of tectochrysin (1, 5 and 10 μg/mL) for 72 h, the cytotoxicity effect of tectochrysin on normal colon cell viability was determined by MTT assay. **c** The colon cancer cells were treated with tectochrysin (1, 5 and 10 μg/mL) for 24 h, and then labeled with TUNEL solution. The green color in the fixed cells marks TUNEL-labeled cells. Total number of cells in a given area was determined by using DAPI nuclear staining (fluorescent microscope). The apoptotic index was determined as the DAPI-stained TUNEL-positive cell number/total DAPI-stained cell number (magnification, 200x). These data are expressed as the mean ± SD of three experiments. *P < 0.05 indicates statistically significant differences from the control group

To assess the inhibitory effect of tectochrysin on cell growth of colon cancer cells (SW480, HCT116), we analyzed cell viability by MTT assay. The cells were treated with varying concentrations of tectochrysin (1, 5, 10 μg/mL) for 24 h. As shown in Fig 1a, tectochrysin inhibited cell growth in colon cancer cells in a concentration-dependent manner. Tectochrysin inhibited SW480 cells growth with IC_50_ value of 6.3 μg/mL and HCT116 cells growth with IC_50_ value of 8.4 μg/mL. Morphologic observation showed that the cells were reduced in size by the treatment of tectochrysin (10 μg/mL) in SW480 cells and HCT116 cells. However, tectochrysin was not cytotoxic in the normal CCD-18co cells in the tested concentration by MTT assay (Fig. 1b). To delineate whether the induction of apoptotic cell death is critical for cell growth inhibition by tectochrysin, we evaluated changes in the chromatin morphology of cells using DAPI staining. To further characterize the apoptotic cell death by tectochrysin, we performed TUNEL staining assays, and then the labeled cells were analyzed by fluorescence microscopy. Apoptotic cells number (DAPI-positive TUNEL stained cells) in SW480 cell was increased to 1 and 58 % by 0 and 10 μg/mL tectochrysin, respectively, and 1 and 54 % by 0 and 10 μg/mL tectochrysin in HCT116 (Fig. 1c).

### Effect of tectochrysin on the expression of apoptosis regulatory proteins and the expression of DRs

**Fig. 2:**
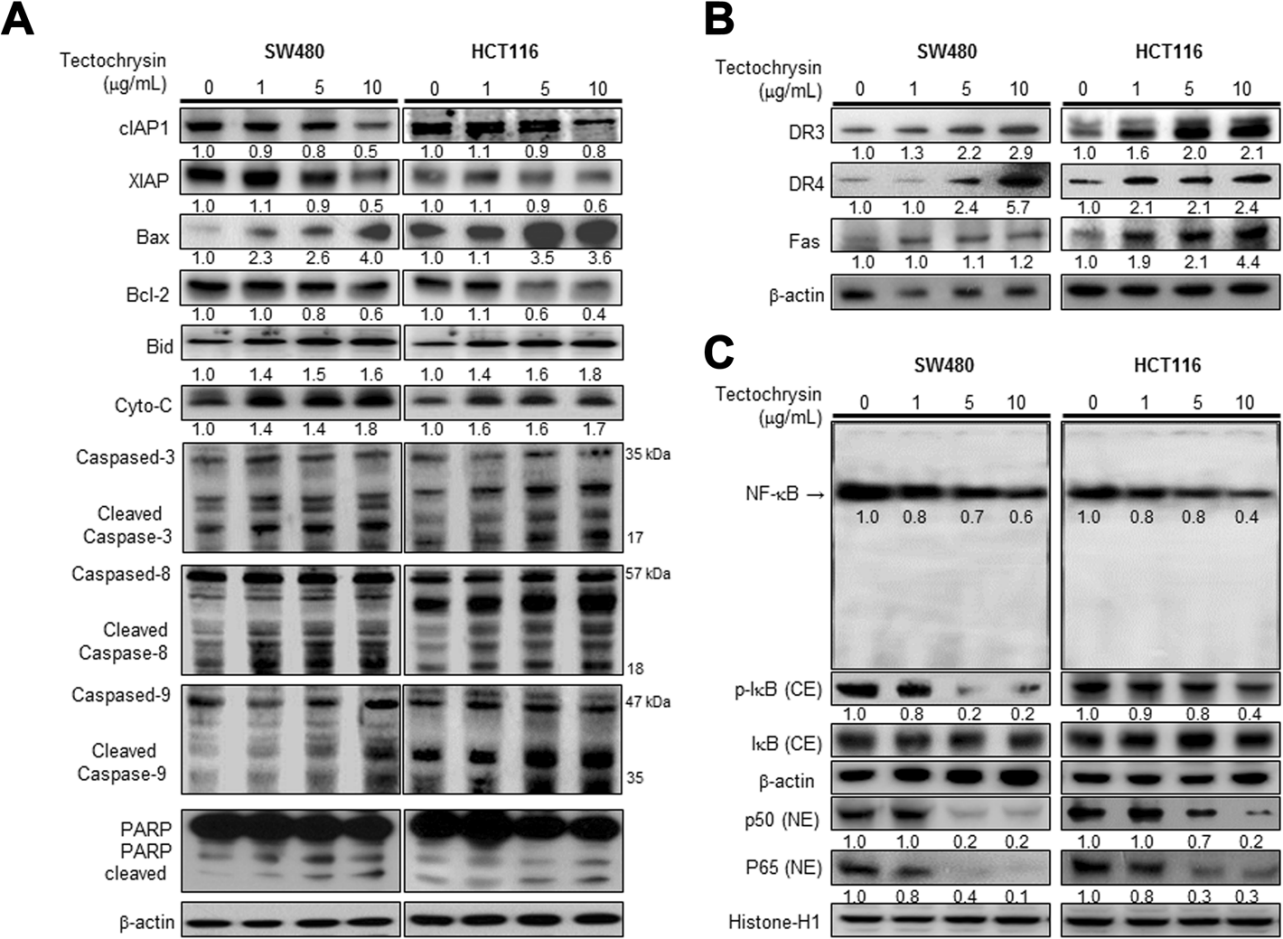
Effect of tectochrysin on the expression of apoptosis regulatory proteins and death receptors, and activation NF-κB in colon cancer cells. The colon cancer cells were treated with different concentrations of tectochrysin (1, 5 and 10 μg/mL) for 24 h or for 1 h (for NF-κB assay). Equal amounts of total proteins (20 μg/lane) were subjected to 10 % or 15 % SDS-PAGE. **a** Expression of cIAP1, XIAP, Bax, Bcl-2, Bid, Cyto-C, cleaved caspase-3, -8, -9, cleaved PARP and β-actin was detected by Western Blotting using specific antibodies. **b** Expression of DR3, DR4, Fas, and β−actin was detected by Western blotting using specific antibodies. **c** Activation of NF-κB was investigated using EMSA as described in Methods. After, the cells were treated with different concentrations (1, 5 and 10 μg/mL) of tectochrysin at 37 °C for 1 h, nuclear or cytosolic proteins were extracted and the expression of p50, p65, IκB-α, and p-IκB-α proteins was detected by Western blotting using specific antibodies. β-actin and histone-H1 proteins were used as internal controls. Each band is representative for three experiments. Values below the band are average of band density. (CE: cytosolic extraction, NE: nuclear extraction)

The relationship between the induction of apoptotic cell death by tectochrysin and the expression of apoptosis related protein was investigated. In agreement with the induction of apoptotic cell death, expression of apoptotic proteins such as cleaved caspase-3, -8, -9, cleaved PARP, Bid, cytochrome-C and Bax was increased, but expression of Bcl-2, cIAP1 and XIAP was decreased in both SW480 and HCT116 colon cancer cells in a concentration dependent manner (Fig. 2a). The extrinsic pathway of apoptotic cell death requires induction of DRs expression. Moreover, expression of DRs is critical for activation of caspase-3. Therefore, we investigated the effect of tectochrysin on the expression of DRs. The expression of DR3, DR4 and Fas was highly induced by tectochrysin in a concentration-dependent manner in both SW480 and HCT116 colon cancer cells (Fig. 2b), but expressions of other DRs were not changed (data not shown). Our data indicated that tectochrysin induced intrinsic apoptosis by elevation of the BAX/BCL2 ratio, cytochromeC and cleaved caspase-3 and-9, and extrinsic pathway by increase of death receptors and cleavage caspase-8. Thus, Tectochrysin may act both intrinsic apoptosis and extrinsic apoptosis pathway.

### Effect of tectochrysin on NF-κB activation

Since highly activated NF-κB is an implicated factor in cell survival as well as in the resistance against therapeutics of colon cancer cells, we determined the inhibitory ability of tectochrysin on the DNA binding activity of NF-κB. We found a higher level of constitutive activation of the NF-κB in the untreated cells. However, the treatment of tectochrysin (1, 5, 10 μg/mL) for 1 h inhibited the constitutively activated DNA binding activity of NF-κB in a concentration-dependent manner. We also found that tectochrysin concentration-dependently inhibited the translocation of p50 and p65 into the nucleus through inhibition of the phosphorylation of IκB (Fig. 2c).

### Abolishing effect of DR3, DR4 and Fas siRNA on the tetochrysin induced growth inhibition, apoptosis, expression of apoptosis regulatory proteins and NF-κB

**Fig. 3:**
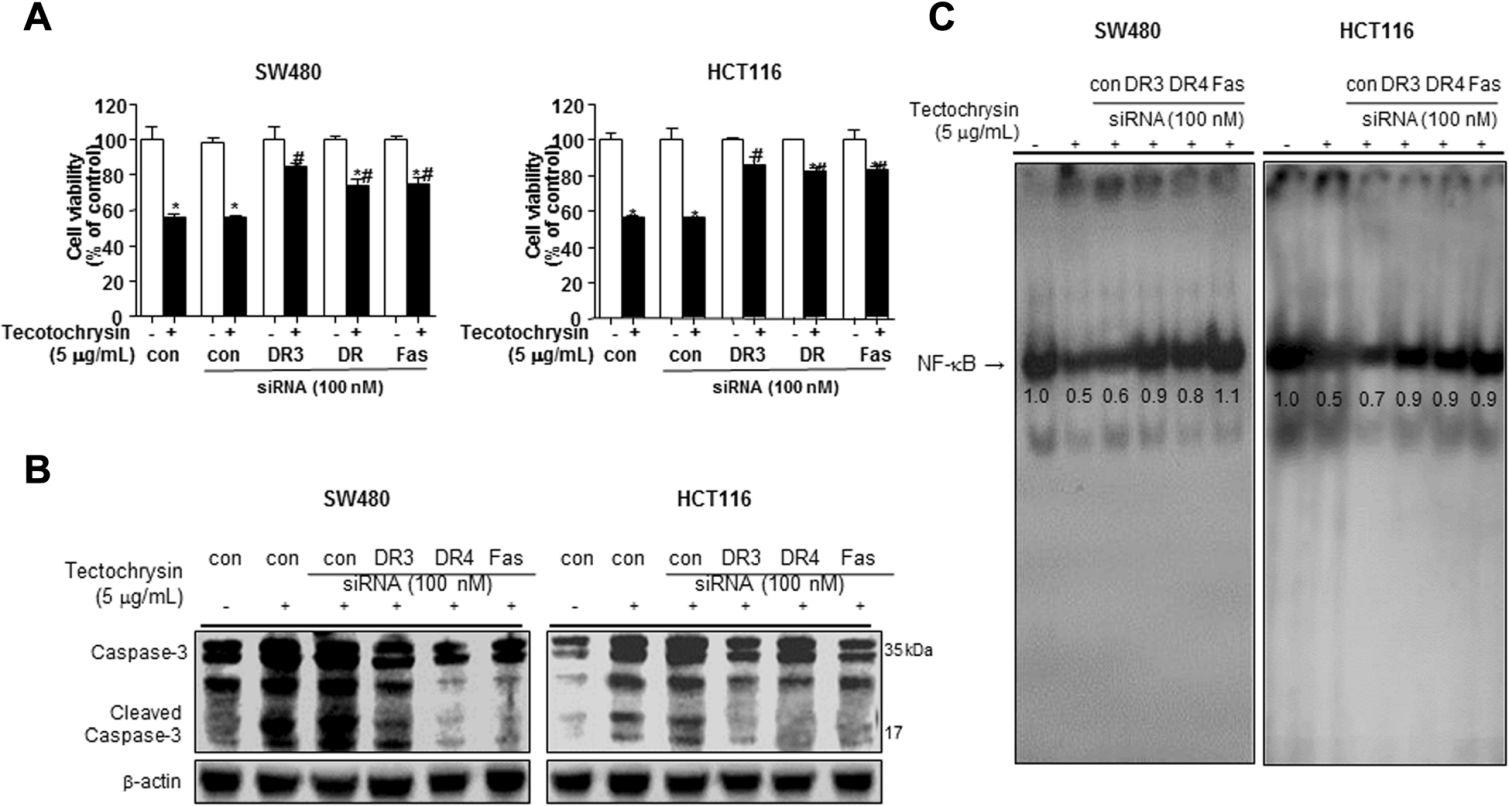
Effect of DRs siRNA on the tectochrysin-induced colon cancer cell growth inhibition, expression of cleaved caspase-3 and NF-κB inactivation. **a** The colon cancer cells were transfected with targeting DR3, DR4, and Fas siRNA (100 nM) for 24 h, and then treated with tectochrysin for 24 h. Then, the cells were determined by MTT assay. The results were expressed as a percentage of viable cells. The data are expressed as the mean ± SD of three experiments. *P < 0.05 compared with the untreated control. ^#^P < 0.05 compared with the treated control. **b** Expression of cleaved caspase-3 and β-actin was detected by Western Blotting using specific antibodies. β-actin proteins were used as internal controls. **c** The activation of NF-κB was investigated using EMSA as described in Methods. Each band is representative for three experiments. Values below the band are average of band density

In order to investigate involvement of DR3, DR4 and Fas in the tectochrysin-induced cancer cell growth, the effects of DR3, DR4 and Fas siRNA on tectochrysin (5 μg/mL)-induced cell growth inhibition was analyzed by MTT assay. Transfection of DR3, DR4, Fas siRNA abolished the cell growth inhibitory effect of tectochrysin on colon cancer cells (Fig. 3a). In agreement with these findings, the decreased expression of cleaved caspase-3 protein by tectochrysin (Fig. 3b) and DNA binding activity of NF-κB by tectochrysin were also abolished in SW480 and HCT116 colon cancer cells treated with DR3, DR4 and Fas siRNA (Fig. 3c).

### Interaction between tectochrysin and NF-κB

**Fig. 4:**
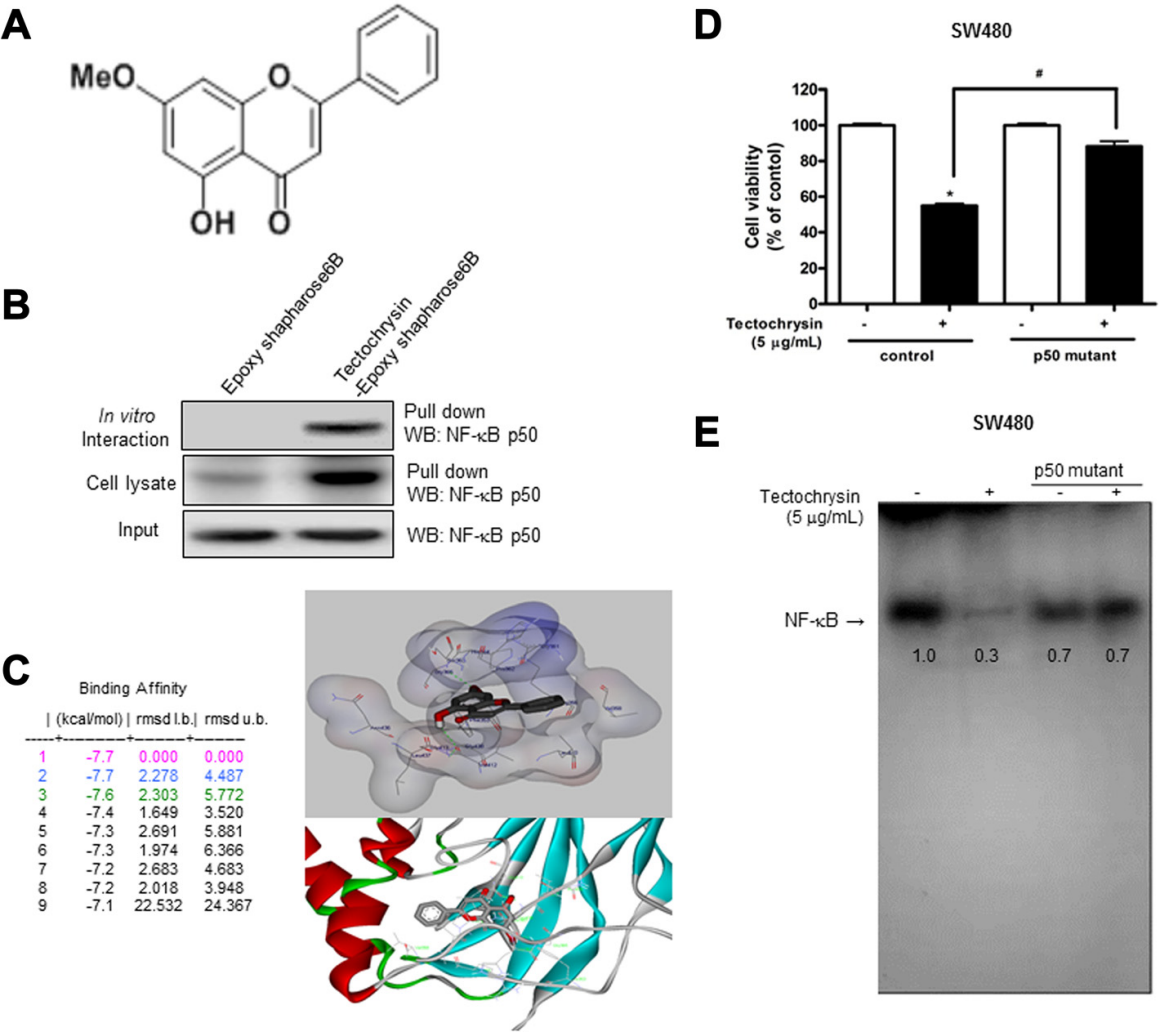
Structural interaction between tectochrysin and NF-κB. **a** Structure of tectochrysin. **b** Pull-down assay identifies an interaction between the tectochrysin and NF-κB p50. Tectochrysin was conjugated with epoxy-Sepharose 6B as described in Methods. **c** Docking model of tecotochrysin with NF-κB p50 as described in Methods. **d** The p50 mutant cells were treated tectochrysin for 24 h, and then analyzed by MTT assay. The data are expressed as the mean ± SD of three experiments. *P < 0.05 compared with the untreated control. ^#^P < 0.05 compared with treated control. **e** SW480 colon cancer cells were transiently transfected with control or mutant types of p50 for 24 h as described in Methods, and then the cells were treated with tectochrysin for 1 h to determine DNA binding activity of NF-κB. Values below the band are average of band density

The interaction of tectochrysin-epoxy Sepharose beads with p50 recombinant protein or cell lysate containing p50 protein was assessed using a pull-down assay. The interaction of tectochrysin-epoxy Sepharose beads with p50 was then detected by immunoblotting with anti-p50 antibody. The results indicated that tectochrysin (Fig. 4a) interacted with recombinant p50 protein or cell lysates containing p50 from SW480 treated with tectochrysin (Fig. 4b). To identify the binding site of tectochrysin to p50, we performed computational docking experiments with tectochrysin and p50. The docking study showed that tectochrysin forms two hydrogen bonds with Gly365 and Val412 of the p50 unit on the amide backbone (binding affinity = -7.6 kcal/mol). It is further surrounded by neighboring hydrophobic amino acid residues such as Val358, Phe353, Ser363, Leu437, and Leu440. Many of these residues are from β-sheets and a loop near DNA binding areas, which may interfere with the DNA binding to the dimeric NF-κB (Fig. 4c). Mutation of Gly365 did not make any difference in binging affinity between tectochrysin and hydrogen bond of p50, but replacing Val412 has a stronger of binding affinity because mutation of this amino acid residue disrupts van Der Waals packing interaction between tectochrysin and p50. Thus, to further determine the direct binding effect of Val412 of p50 with tectochrysin, p50 mutant plasmid (Vp50A) was transfected into SW480, and checked cell growth and NF-κB activity. As expected, tectochrysin did not inhibit the growth of colon cancer cell transfected with mutant p50 (Fig. 4d). EMSA also showed that tectochrysin did not completely inhibit NF-κB activity (Fig. 4e). These results clearly suggested that tectochrysin mediates its effects through binding to Val 412 residue of the p50 subunit of NF-κB.

### Combination effect of tectochrysin and TRAIL in TRAIL-resistant cancer cell growth

**Fig. 5:**
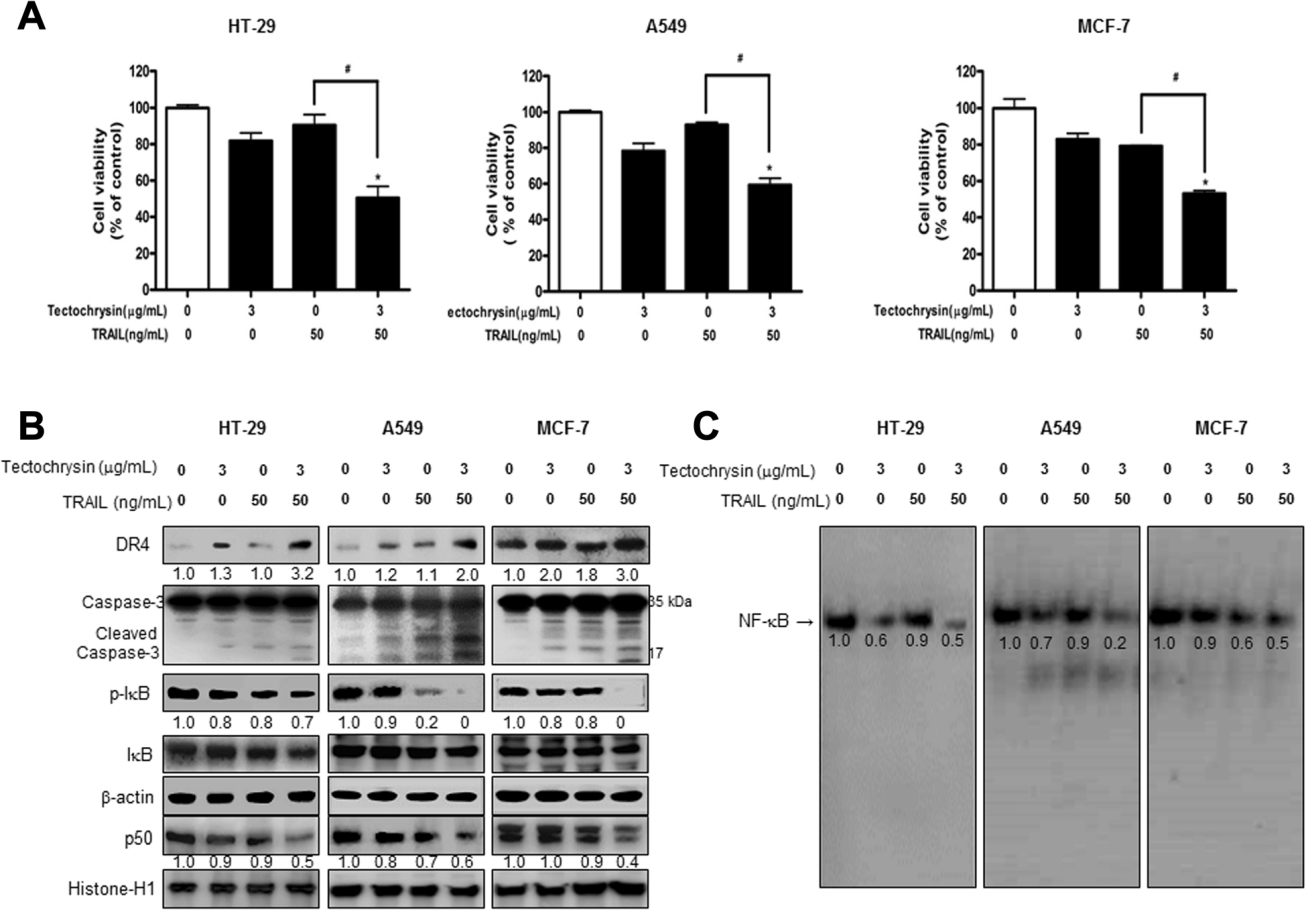
Effect of tectochrysin on cell growth, DR4 and cleaved caspase-3 expression, and NF-κB activity in TRAIL-resistant cancer cell. **a** HT-29, A549, and MCF-7 cells were pretreated with tectochrysin (3 μg/mL) for 24 h, the media were removed, and the cells were exposed to TRAIL (50 ng/mL in HT-29, A549, and MCF-7 cells) for 24 h. The effect of tectochrysin on resistant cancer cell growth was determined by MTT assay. The data are expressed as the mean ± SD of three experiments. *P < 0.05 compared with the control. ^#^P < 0.05 compared with the treated TARIL treated cells. **b** Expression of DR4, p-IκB, IκB, p50, β-actin and histone-H1 was detected by Western blotting using specific antibodies. β-actin and histone-H1 proteins were used as internal controls. **c** The activation of NF-κB was investigated using EMSA as described in Methods. Each band is representative for three experiments. Values below the band are average of band density

The human colon cancer cell lines SW480 and HCT116 are known to be TRAIL-sensitive, while HT-29 (human colon cancer cell), A549 (human lung cancer cell) and MCF-7 (human breast cancer cell) are TRAIL-resistant [26]. The aim of this study was to investigate whether tectochrysin can enhance the sensitivity of TRAIL resistant cancer cells to TRAIL and possible mechanisms. We treated these cancer cells with half a dose of IC_50_ tectochrysin (3 μg/mL) and TRAIL (50 ng/mL), and we found that tectochrysin and TRAIL treatments alone induced 18.1 % and 9.6 % cell growth inhibition in HT-29 cells, 21.7 % and 7.1 % in A549 cells and 17.1 % and 20.8 % in MCF-7 cells. However, a combination treatment with tectochrysin and TRAIL enhanced TRAIL induced cell growth inhibition up to 49.6 % in HT-29 TRAIL-resistant colon cancer cells, 40.7 % in A549 TRAIL-resistant lung cancer cells and 46.8 % in MCF-7 TRAIL-resistant breast cancer cells (Fig. 5a). The combination index values of A549 and HT-29 cells were 0.021, and MCF-7 cells was 0.034. These data indicate that tectochrysin leads to a synergistic growth inhibitory effect in TRAIL-resistant cancer cells with TRAIL. In addition, we compared the effect of tectochrysin and/or TRAIL of the activation of caspase-3 and the expression of DR4 in the TRAIL-resistant cancer cells. Although tectochrysin and TRAIL alone had little effect on activation of caspase-3 cleavage and DR4 expression, the combination treatment significantly increased expression of cleaved caspase-3 and DR4 (Fig. 5b). Tectochrysin also further reduced DNA binding activity of NF-κB as well as translocation of p50 in the nucleus and cytosolic p-IκB expression in TRAIL-resistant cancer cells (Fig. 5c).

### Effect of tectochrysin on the colon tumor growth in in vivo xenograft model

**Fig. 6:**
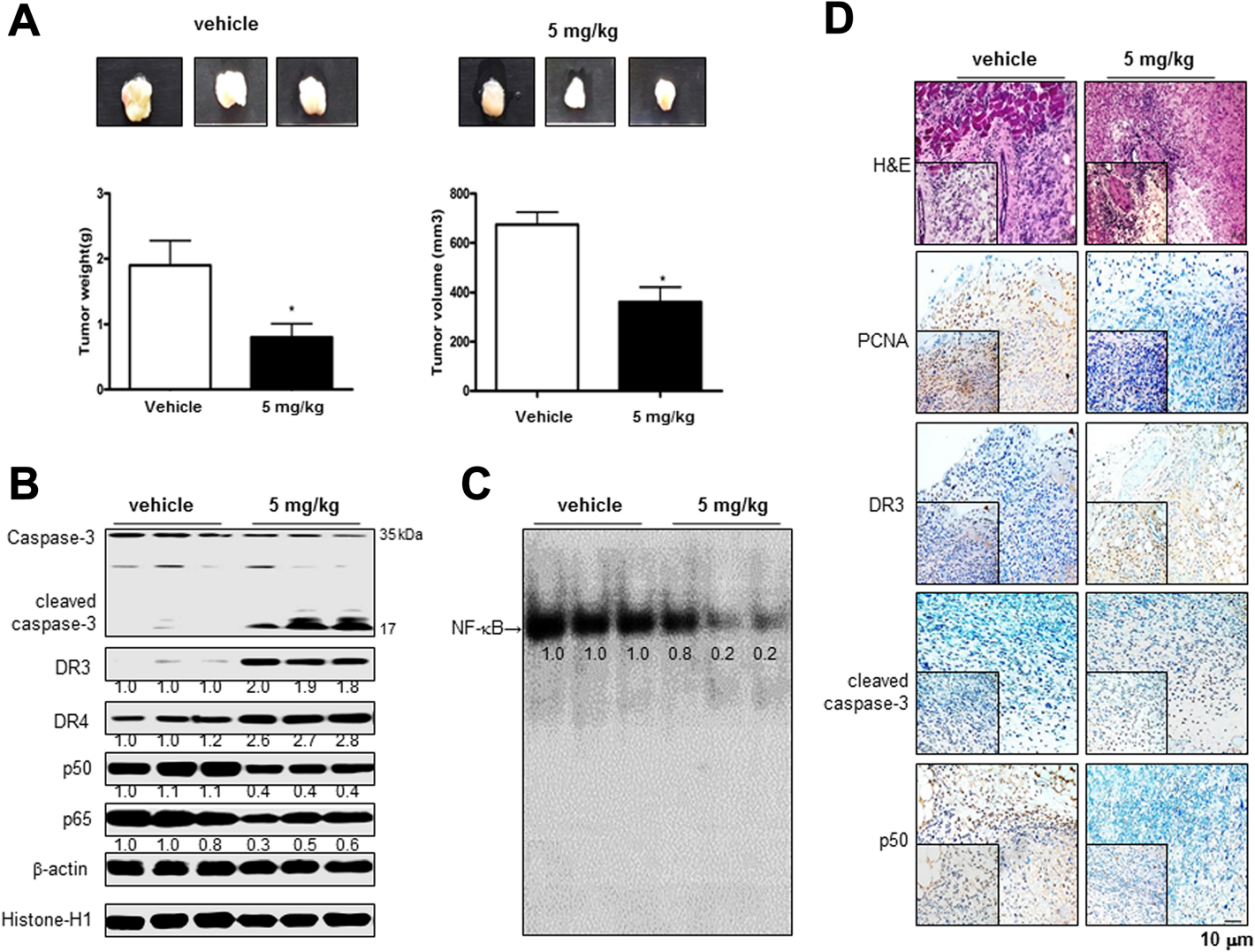
Effect of tectochrysin on the tumor growth in HCT116 xenografts in vivo model. HCT116 xenografts mice were i.p treated with tectochrysin (5 mg/kg twice a week) for 3 weeks. **a** Tumor images (left, vehicle; right, tectochrysin 5 mg/kg), weights and volumes. The data are expressed as the mean ± SD of ten mice. *P < 0.05 compared with the vehicle mice. **b** Tumor extracts were analyzed by Western boltting. Samples (20 μg/lane) were resolved on 10 % or 15 % SDS-PAGE and detected with antibodies against cleaved caspase-3, DR3, DR4, p50, p65, β-actin, and Histone-H1. β-actin and histone-H1 proteins were used as internal controls. **c** DNA binding activity of NF-κB was determined by EMSA in nuclear extracts from tumor tissue of mice, as described under Methods. **d** Immunohistochemistry was used to determine expression levels of H&E, PCNA, DR3, cleaved capase-3, and p50 in tumor tissue of mice as described in Methods. Each figures represent for three animals, and each band is representative for three experiments. Values below the band are average of band density

To elucidate the antitumor effects of tectochrysin in *in vivo*, the tumor growth in colon cancer xenograft–bearing nude mice following tectochrysin treatments was investigated. In HCT116 xenograft studies, tectochrysin was administrated i.p. twice per week for 3 weeks to mice with tumors ranging from 200 to 300 mm^3^ in volume. The mice were weighed twice per week. The changes in body weights between the control and the tectochrysin-treated mice (n = 10) were not remarkably different during the experiment (data not show). However, Fig. 6a showed the relative tumor growth delay measured after the treatment of tectochrysin in comparison with the vehicle group. On day 21, the final tumor weights were recorded. Tumor weights and volumes in mice treated with tectochrysin at 5 mg/kg doses were 57.9 % and 46.4 % of the vehicle group, respectively (Fig. 6a). In agreement with the in vitro results, expression of cleaved caspase-3, DR3 and DR4 was significantly increased in the tumor tissues treated with tectochrysin (Fig. 6b). DNA binding activities of NF-κB and translocation of p50 and p65 into nucleus were clearly lowered in tumor tissues treated with tectochrysin (Fig. 6b and c). The immunohistochemical analysis of tumor sections by H&E and by proliferation antigens against PCNA, cleaved caspase-3 and DR3 staining revealed that tectochrysin inhibited tumor growth (Fig. 6d).

## Discussion

Recent evidence indicates that NF-κB signaling pathway is significantly involved in tumor development [27]. The constitutively activated NF-κB transcription factor has been associated with several aspects of tumorigenesis such as tumor cell growth, anti-apoptosis, metastasis, angiogenesis, resistance against chemotherapeutics, and tumor promotion in many cancer cells including colon cancer [28]. The NF-κB transcription factor is constitutively activated in human colorectal carcinoma tissue and colon cancer cells to give favorable circumstance for cancer cell growth [12, 29]. There have been many recent reports demonstrating anti-cancer effects of several compounds through inactivation of NF-κB. Our previous studies also showed that compounds inhibiting NF-κB activity such as 4-O-methylhonokiol and inflexinol inhibited colon cancer cell growth [30, 31, 14, 13]. Several studies have especially showed that flavonoids inhibit colon cancer growth through inactivation of NF-κB. For example, Methyl 3,5-dicaffeoyl quinate (200 μg/mL), a flavonoid glucoside, induced cell cycle arrest and apoptosis by inhibiting NF-κB activation in HT-29 human colon cancer cells [32]. Furthermore, the flavonoids inhibited other cancer growth via inactivation of NF-κB. Quercetin, a principal flavonoid compound in onions, inhibits human oral cancer cells through inhibition of NF-κB [33]. We, in the present study, found that tectochrysin inhibited constitutively activated NF-κB in colon cancer cells and colon cancer tissues in xenograft animal model. In *in vitro*, tectochrysin prevented NF-κB target anti-apoptosis gene expression (but increased apoptosis gene expression) and inhibited DNA binding activity of NF-κB. In *in vivo*, the immunohistochemical analysis of tumor section by p50 staining revealed that translocation of p50 into nucleus was significantly lowered in the tumor tissues treated with tectochrysin. We also found that tumor tissues treated with tectochrysin inhibited the activated DNA binding activity of NF-κB. Thus, it is possible that alteration in the expression level of NF-κB target anti-apoptotic and pro-apoptotic proteins is likely to influence apoptotic cell death by tectochrysin. These data indicates the possibility that tectochrysin may inhibit NF-κB, thereby inhibit colon cancer cell growth. Thus, tectochrysin, like other flavonoid compounds, could be effective for the treatment of colon cancer cells.

In further chemical target identification studies, the interaction of tectochrysin-epoxy-sepharose 6B beads with NF-κB p50 protein was assessed using a pull-down assay. The interaction of tectochrysin-epoxy-sepharose 6B beads with NF-κB p50 was then detected by immunoblotting with anti-NF-κB p50 antibody. The results indicated that tectochrysin interacted with NF-κB p50. To identify the binding site of tectochrysin to NF-κB p50, we performed computational docking experiments with tectochrysin and NF-κB p50. Tectochrysin forms two hydrogen bonds with Gly365 and Val412 of the p50 subunit on the amide backbone. It is further surrounded by neighboring hydrophobic amino acid residues such Val358, Phe353, Ser363, Val412, Leu437 and Leu440. Many of these residues are from β-sheets and a loop near DNA binding areas, which may interfere with the DNA binding to the dimeric NF-κB. Our previous studies reported that inflexinol and snake venom toxin modifies a cysteine residue and cysteine62 of p50 in NF-κB via direct interaction of these sites, and thus inhibited the DNA binding activity of NF-κB [14, 34]. Another study also showed that kaurane diterpene inhibited NF-κB directly targeting the DNA-binding activity of cysteine62 in p50 [35]. Moreover, the abolished effect on growth inhibition and inactivation of NF-κB in colon cancer cells transfected a p50 mutant (Vp50A, Valine412 was substituted with Alanine) were observed after a treatment with tectochrysin. Therefore, these data indicate that tectochrysin inhibits colon cancer cell growth through inactivation of NF-κB by direct binding on Val412 residue of p50.

DRs are the cell surface receptors that are a part of tumor necrosis factor (TNF) members of cytokines. It is well-known that apoptosis can be induced by stimulation of DRs including TNFR1/2, DR3, DR4, DR5, DR6 and Fas by their respective ligands [36]. Therefore, these receptors emerged as attractive targets for anti-cancer therapeutics. Several compounds induced apoptotic cell death of cancer cells through increasing DR expression. Our previous study showed that snake venom toxin induced apoptosis of HCT116 and HT-29 colon cancer cells via enhancement of DR4 and DR5 expression [26]. Flavonoid, such as fucoidan, increased apoptosis of human colon cancer cells via increased expression of DR4, DR5 and Fas [37]. Flavonoid compounds are also known to have an anti-cancer effect through activation of DR-caspase pathways. Caspases play a critical role in apoptosis by DRs [38]. Hesperetin, a flavonoid from citrus fruits, exhibited a potential anticancer activity against human cervical cancer cell lines through the induction of apoptosis via caspase-3 activation by increasing Fas expression [39]. Genistein, one of well-known isoflavones, enhanced apoptosis in lung cancer cells induced by increasing the expression of cleaved caspase-3 via up-regulation of TNFR-1 DR signaling [40]. Other compounds such as grape seed extract inhibited human colon cancer cell growth by increasing caspase-3 [41]. Similar to these results, our results showed that the expression of DR3, DR4, Fas and cleaved caspase-3 and -9 highly increased by tectochrysin in a concentration-dependent manner. Moreover, knock down of DR3, DR4 and Fas with siRNA abolished the growth inhibitory effect and caspase-3 activation of tectochrysin on colon cancer cells. In a previous study, we found that tectochrysin inhibited lung cancer cell growth via overexpression of DR3 and Fas [25]. These data demonstrated that depending on the different cancer type, differential DR pathway may be significant. These results indicate that the colon cancer cell growth inhibitory effects of tectochrysin could be related caspase-3 pathway linked to DR3, DR4 and Fas.

NF-κB inhibition could also be effective to overcome chemo-resistance [42]. Our previous study reported that natural snake venom toxin suppressed TRAIL-resistant HT-29, A549 and HepG2 cells growth via inhibition of NF-κB activity [26, 43] . Our present results showed that tectochrysin further inhibited TRAIL-inactivated NF-κB activity, as well as expression of p50 and p-IκB in HT-29 (resistant colon cancer cell), A549 (resistant lung cancer cell) and MCF-7 (resistant breast cancer cell). Therefore, tectochrysin inhibits colon cancer cell growth and has the potential to overcome chemotherapy resistance. TRAIL is a potential anticancer agent because of its capacity to kill selectively cancer cells without toxic effects on normal cells, and thus many chemoresistant cancer cells are resistant to TRAIL [44]. Previous studies have reported that natural compounds enhanced TRAIL induced apoptotic cell death in TRAIL-resistant cancer cells. Curcumin, a natural flavonoid compound, can synergistically induce apoptosis in three TRAIL-resistant breast cancer cell lines [45]. We found that tectochrysin enhanced TRAIL induced cell growth inhibition up to 65.8 % in HT-29 TRAIL-resistant colon cancer cells. We also demonstrated that the synergistic effects of tectochrysin and TRAIL on the activation of caspase-3 cleavage and the expression of DR4 in the TRAIL-resistant cancer cells (HT-29, A549 and MCF-7). Combination index values of A549 and HT-29 cells were 0.021, and MCF-7 was 0.034. These results indicated that tectochrysin enhances TRAIL-induced apoptotic cell death through the over-expression of DR4 as well as the down-regulation of anti-apoptotic protein expression via inhibiting NF-κB pathways.

In a xenograft nude mouse, tumor weight and volume in mice treated with tectochrysin at 5 mg/kg doses were 57.9 % and 46.4 % of the vehicle group, respectively. The expression of DR3, DR4 and cleaved caspase-3 was also significantly increased in tectochrysin treated mice, but DNA binding activities of NF-κB and translocation of p50 and p65 into the nucleus were clearly lowered in tumor tissues treated with tectochrysin. Silibinin and wogonin, different flavonoids, inhibited HCT116 colon cancer cells with IC_50_ value of 75 μg/mL and 42.6 μg/mL [46, 47]. However, in the present study, tectochrysin inhibited human colon cancer cells growth with IC_50_ value of 8.4 μg/mL and 6.3 μg/mL. In *in vivo* study, silibinin (200 mg/kg) or aciculatin (30 mg/kg), inhibited human colon tumor growth about 49.1 %, 40 % respectively [48, 49]. However, 5 mg/kg tectochrysin showed 48.1 % inhibition in HCT116 human colon cancer growth. These data indicate that tectochrysin could be more for chemotherapeutics compared to other flavonoids. Moreover, we also found that tectochrysin could be a well absorbed compound as a high degree of plasma protein binding compound as determined by the ADME prediction program (pre ADME version 1.0.2). Several drug-likeness predictions such as Lipinski’s, Lead-like, CMC-like, 2.91 as sklogP value and WDI-like rules indicate that this compound is suitable to be used as a drug. Toxicity prediction indicated that there is no toxic effect by this compound. In conclusion, the current study showed that tectochrysin exerts its cell growth inhibitory effects through inhibition of NF-κB and enhancement of DR expression in human colon cancer cells, and enhances sensitivity of TRAIL-resistant cancer cells, suggesting that tectochrysin can be a useful agent for the treatment of colon cancer as well as an adjuvant agent for chemo-resistant cancer.

## Methods

### Chemicals

We subsequently identified the key compound according to activity-guided purification, as described elsewhere [25]. The active principle was obtained as white amorphous powder with physico-chemical properties of ESI-MS *m/z*: 291 [M + Na]^+^; ^1^H-NMR (500 MHz, CDCl_3_): ,^13^C-NMR (100 MHz, CDCl_3_). The structure of tectochrysin was identified by comparison with its physico-chemical and spectroscopic data reported by an investigator [50] as described elsewhere [25].

### Cell culture

SW480, HCT116, HT-29, A549 and MCF-7 cells were obtained from the American Type Culture Collection (Manassas, VA). SW480, HCT116, HT-29, A549 and MCF-7 cells were cultured in RPMI 1640 and DMEM medium supplemented with 10 % fetal bovine serum (FBS) and penicillin/streptomycin (100 U/mL). Cell cultures were then maintained at 37 °C in a humidified atmosphere with 5 % CO_2._ The human colon CCD-18co normal cell was also obtained from the Korea Cell Line Bank and were grown in DMEM medium with 10 % fetal bovine serum, 25 mM HEPES and penicillin/streptomycin (100 U/mL) at 37 °C in a humidified atmosphere with 5 % CO_2_.

### MTT assay and evaluation of apoptotic cell death

Each cell line ($1\times {10}^4$ cells) was incubated in 200 μl of RPMI 1640, DMEM medium with tectochrysin (concentrations ranging from 1, 5, 10 μg/mL) in a 96-well flat-bottomed plate in triplicate. After incubation for 72 h at 37 °C, MTT (3-(4,5-dimethylthiazol-2-yl)-2,5-diphenyltetrazolium bromide; Sigma, St Louis, MO, USA) diluted in RPMI 1640, DMEM medium were added to each well and incubation was carried out for 90 min. The supernatant was then discarded and the crystal products were eluted with DMSO (200 μL/well; Sigma, St Louis, MO, USA). Colorimetric evaluation was performed with a spectrophotometer at 540 nm. The apoptosis assay was first performed by using DAPI staining. SW480 and HCT116 human colon cancer cells were cultured with concentrations of tectochrysin (5 μg/mL), and induction of apoptotic cell death was evaluated after 24 h. Tunel assay was done as described previously [51].

### Western blot analysis and gel electromobility shift assay

Western blot analysis was performed as described previously [25]. The membranes were immunoblotted with the following primary antibodies: mouse monoclonal antibodies directed against Fas, Bax, p65, p-IκB, cytochrome-C, β-actin, and Histone-H1 (1:1000 dilutions; Santa Cruz Biotechnology), and rabbit polyclonal antibodies directed against DR3, DR4, Bid, p50, and IκB (1:1000 dilutions; Santa Cruz Biotechnology), and against c-IAP1, XIAP, Bcl-2, cleaved caspase-3, -8, -9 and PARP (1:1000 dilutions; Cell Signaling Technology, Beverly, MA). Immunoreactive proteins were detected with the Enhanced Chemiluminescence Western blotting detection system (Amersham Pharmacia Biotech, Inc., Buckinghamshire, UK). Gel electromobility shift assay (EMSA) was done as described previously [25]. The relative density of the protein bands was scanned by densitometry using My Image and quantified by Labworks 4.0 software (UVP, Inc., California, USA).

### Transfection

Colon cancer cells (SW480, HCT116 $7\times {10}^3$ cells/well) were plated in 96-well plates and transiently transfected with 0.4 μg of the empty vector or the constitutively activated 100 nM of negative siRNA, DR3, DR4 or Fas siRNA per well, using a mixture of plasmid and the WelFect-EX PLUS reagent in OPTI-MEM, according to the manufacturer’s specification (WelGENE, Seoul, Korea). The p50 mutant (Vp50A, valine 412 was substituted with Alanine) plasmid was also transfected with welfect-EX plus reagent in OPTI-MEM according to the manufacturer׳s specification (WelGENE, Seoul, Korea). DR3 siRNA seq. 5’-GAAGCCCUAAGUACGGUUAtt; DR4 siRNA seq. 5’-CUCUGAUGCUGUUCUUUGAtt; Fas siRNA seq. 5’ -GAACCCGUGUUUGCAAUCAtt.

### Pull-down assay

Western blot analysis was performed as described previously [25]. The cell lysate or NF-κB (p50) recombinant protein (Abnova, Taipei, Taiwan) were mixed with tectochrysin-conjugated Sepharose 6B or Sepharose 6B at 4 °C for 24 h. The beads were then washed three times with TBST. The bound proteins were eluted with SDS loading buffer. The proteins were then resolved by SDS-PAGE followed by immunoblotting with antibodies against NF-κB p50 (1:1000 dilution, Santa Cruz Biotechnology).

### Docking procedure for NF-κB with tectochrysin

Molecular docking studies were performed using Autodock VINA. NF-κB was obtained from the X-ray crystal structure of NF-κB p50/p65 heterodimer complexed to the immunoglobulin κB DNA. (PDB ID: 1VKX). Only p50 of the heterodimer NF-κB structure of p50/p65 was used in the docking experiments and conditioned using AutodockTools by adding all polar hydrogen atoms. The grid box was centered on the p50 and the size of the grid box was adjusted to include the whole monomer. Docking experiments were performed at various exhaustiveness values of the default, 24, and 48. After the best binding mode was chosen, another round of docking experiments were performed with the grid box re-centered at the binding site of the best ligand-binding mode with its grid box size of 30 × 30 × 30.

### Antitumor activity study *in vivo* xenograft animal model

Five-week-old male BALB/c athymic nude mice (n = 10/group) were purchased from Japan SLC, Inc. (Shizuoka, Japan) and housed in clean specific pathogen free (SPF) rooms. All experiments were approved and carried out according to the Guideline for the Care and Use of Animals of the Chungbuk National University Animal Care Committee (CBNU-278-11-01). HCT116 cancer cells were injected subcutaneously (1 × 10^7^ cells/0.1 mL PBS/animal) into the lower right flanks of mice. After 14 days, when the tumors had reached an average volume of 200–300 mm^3,^ the tumor-bearing nude mice were intraperitoneally injected with tectochrysin (5 mg/kg dissolved in 0.1 % DMSO) twice per week for 3 weeks. In *in vitro* experiments, the IC_50_ value of 8.4 μg/mL in HCT116 appeared, thus the concentration of the drug (5 mg/kg) was set in animal models. The tumor volumes were measured with vernier calipers and calculated by the following formula: (A × B^2^)/2, where A is the larger and B is the smaller of the two dimensions.

### Immunohistochemistry

All specimens were fixed in formalin and paraffin-enclosed for examination. Sections 4 μm thick were stained with Hematoxylin and Eosin (H&E) and immunohistochemistry as described elsewhere [14].

### Data analysis

The data were analyzed using the GraphPad Prism 4 ver. 4.03 software (GraphPad Software, La Jolla, CA). Data are presented as mean ± SD. The differences in all data were assessed by one-way analysis of variance (ANOVA). When the P value in the ANOVA test indicated statistical significal significance, the differences were assessed by the Dunnett’s test. A value of P < 0.05 was considered to be statistically significant.

## Abbreviations

NF-κB: Nuclear factor kappa B
DR: Death receptor
RPMI: Roswell Park Memorial Institute medium
DMEM: Dulbecco’s modified Eagle medium
DMSO: Dimethyl sulfoxide
HEPES: 4-(2-hydroxyethyl)-1-piperazineethanesulfonic acid
MTT: 3-(4,5-dimethylthiazol-2-yl)-2,5-diphenyltetrazolium bromide
DAPI: 4’,6-diamidino-2-phenylindole
TUNEL: Terminal deoxynucleotidyl transferase dUTP nick end labeling
PARP: Poly ADP ribose polymerase
EMSA: Gel electromobility shift assay
siRNA: Small interfering RNA
TRAIL: TNF-related apoptosis-inducing ligand
PCNA: Proliferating cell nuclear antigen
